# Whole-genome sequence of a genotype VIII infectious bronchitis virus isolated from California layer chickens in 2021

**DOI:** 10.1128/MRA.00959-22

**Published:** 2023-10-17

**Authors:** Rachel Jude, Ana P. da Silva, Daniel Rejmanek, Beate Crossley, Carmen Jerry, Simone Stoute, Rodrigo Gallardo

**Affiliations:** 1 Department of Population Health & Reproduction, University of California Davis, Davis, California, USA; 2 California Animal Health & Food Safety Lab, University of California Davis, Davis, California, USA; 3 California Animal Health & Food Safety Lab, University of California Davis, Turlock, California, USA; Katholieke Universiteit, Leuven, Belgium

**Keywords:** genotype VIII, infectious bronchitis virus, poultry

## Abstract

Herein, we report the complete genome for an avian infectious bronchitis virus isolated from cecal tonsils of California layers in 2021. This whole-genome sequence belongings to genotype GVIII, previously classified as a unique variant.

## ANNOUNCEMENT

Avian infectious bronchitis virus (IBV), of genus *Gammacoronavirus* and family *Coronaviridae*, causes a contagious, endemic disease (infectious bronchitis, IB) in chickens associated with respiratory and urogenital consequences and severe economic losses (1 – 3). Its genome is an approximately 27 kb positive-sense, single-stranded RNA encoding for two polyproteins, four structural proteins, and four accessory proteins. A recently introduced IBV classification system using the S1 portion of the spike gene identified 6 genotypes with 32 lineages (4). Since, additional genotypes have been identified using this classification scheme (3, 5, 6). Here, we report the whole-genome sequence of an IBV isolate from California layers belonging to genotype GVIII.

In late 2021, two cecal tonsils collected from 33- and 34-week-old (designated IBV/Ck/USA/CA/21-1883 and IBV/Ck/USA/CA/21-1926, respectively) white Leghorn layers at a California farm experiencing increased mortality and decreased egg production were submitted to the California Animal Health and Food Safety Laboratory. The tissues were IBV positive by RT-qPCR (7), and the virus was amplified in embryonated SPF eggs as previously described (8). Allantoic fluid was ultra-centrifuged (50,000 *g*, 45 min) and reconstituted in 200 µL VTM. Total RNA was extracted from the allantoic fluid using the MagMax-96 Viral Isolation Kit (ThermoFisher, USA). ds-cDNA was generated using the total RNA, random hexamers, and the Maxima H Minus Double-Stranded cDNA Synthesis kit (ThermoFisher) and then purified using AMPure XP beads (1:1) (Beckman Coulter, USA). A library was generated using the ONT Ligation Sequencing Kit [Oxford Nanopore Technologies (ONT), UK]. The cDNA was end-repaired using the NEBNext Ultra II DNA End Prep and Repair kit (New England Biolabs, USA), bead purified (1:1), and eluted in nuclease-free H_2_O. NEBNext Quick T4 DNA ligase (New England Biolabs) ligated AMX-sequencing adapters (ONT) to the cDNA. Adapter-ligated DNA was bead purified (1:2.5), washed twice with short fragment buffer (ONT), and eluted in elution buffer (ONT). Each library was loaded individually onto Flongle flow cells R9.4.1 on a GridION sequencer (ONT) and run for 24 h with super-accuracy basecalling and Q10+ read quality. IBV/Ck/USA/CA/21-1883 returned 35,117 reads with >500X IBV genome coverage and IBV/Ck/USA/CA/21-1926 returned 80,179 reads with >1,000X coverage.

**Fig 1:**
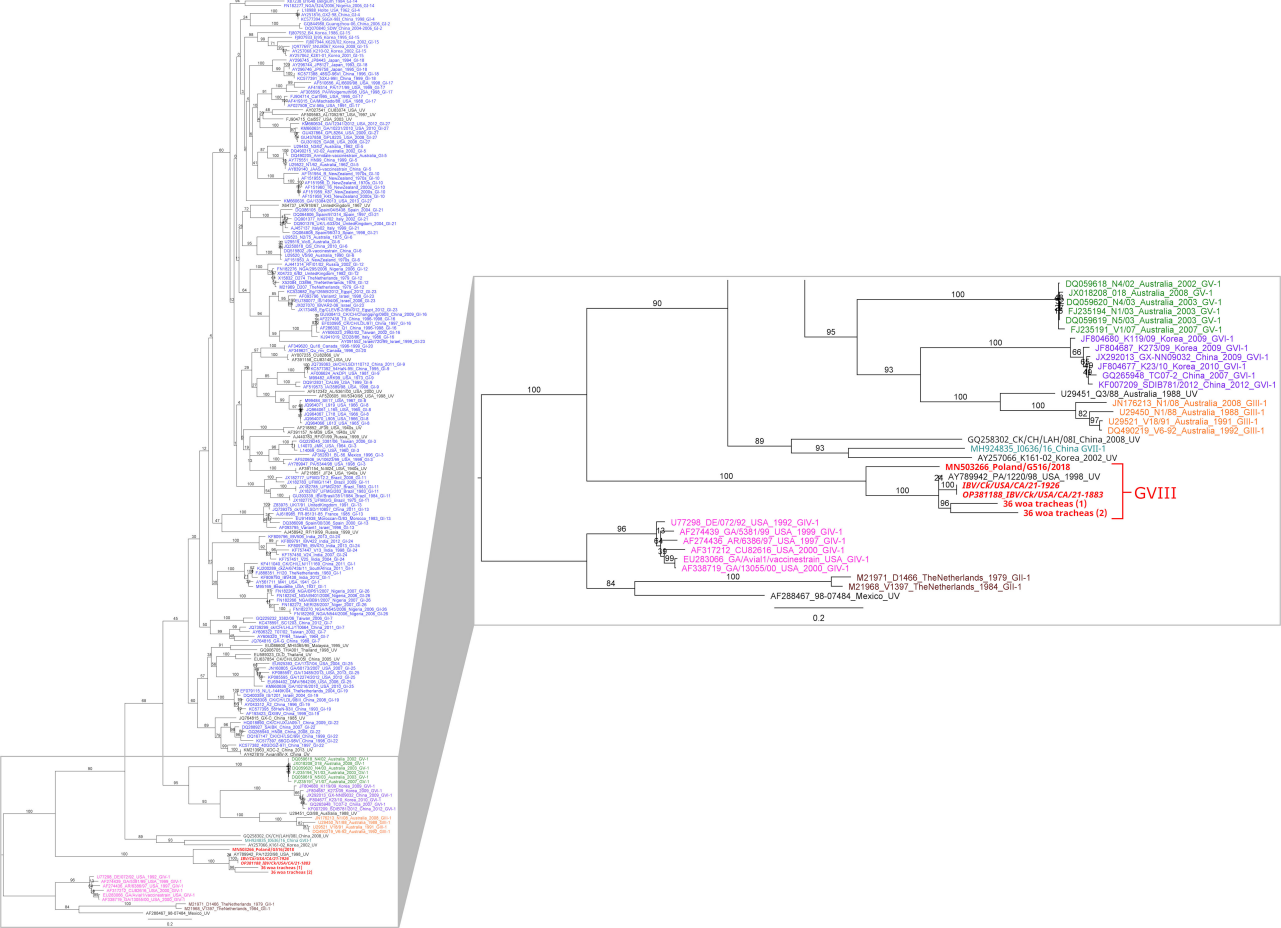
Phylogenetic analysis of the S1 HVR for all known genotypes and lineages. Full-genome sequences were trimmed to the S1 HVR (MW556742, nucleotide positions 20,368–21,119), and phylogenetic analysis was performed using the Geneious Prime RaxML plugin (12) with the GTR GAMMA I nucleotide model; 1,000 bootstrap replicates; and rapid bootstrapping algorithm with search for best-scoring ML tree. Tips are labeled with GenBank accession numbers, descriptors of the strains, and genotypes if information was available. Next to each branch, bootstrap support values represent the percentage of replicate trees grouping sequences in the same branch across 1,000 replicate trees. Branch lengths are scaled by number of substitutions by site. Colors are representative of different genotypes. The zoomed-in portion indicates all genotypes excluding GI (blue). Sequences belonging to genotype GVIII are indicated in red and include S1 HVR sequences of isolates from this study (italicized) as well as sequences of isolates from Poland (MN503266) and Arizona (36 woa tracheas).

Bioinformatics were performed in the Geneious Prime suite 2022.1.1 (Biomatter Ltd.). Reads were mapped using MiniMap2 (9) to strain Beaudette (NC_001451) using default parameters. The complete genomes (full 5′ and 3′ UTRs confirmed by BLAST analysis) of both isolates were identical, 27,668 bp in length, and had a 38.0% GC content. Full genome and S1 HVR (MW556742, nucleotide positions 20,368–21,119) BLAST searches were performed and the highest pairwise identities were 96.7% to GA 1998 (GQ504723) and 96.3% to PA/1220/98 (AY789942), respectively. Partial S1 HVR sequences were aligned in MAFFT (10) using sequences of 199 representative strains (4) and recently identified genotype VII sequences, MN503266 from Poland (6) and in-house sequences from Arizona, USA [36 woa tracheas (1) and (2, 11)]. The sequences of isolates from this analysis as well as the Poland and Arizona sequences were grouped distinctly with PA/1220/98, previously reported as a unique variant (4) but redesignated as genotype GVIII in 2020 (Fig. 1) (6).

## Data Availability

This whole-genome project for Avian coronavirus isolate IBV/Ck/USA/CA/21-1883 genotype GVIII has been deposited in GenBank under the accession no. OP381188. Raw reads have been deposited in the SRA under BioSample accession no. SAMN35989168. The version described in this paper is the first version.
